# Open Pelvic Fracture with Rectal Laceration: A Case Report

**DOI:** 10.31729/jnma.5270

**Published:** 2021-08-31

**Authors:** Sijan Bhattachan

**Affiliations:** 1 Department of Orthopaedics and Trauma Surgery, National Trauma Center, National Academy of Medical Sciences, Kathmandu, Nepal

**Keywords:** *external fixator*, *faecal diversion*, *open pelvic fracture*, *rectal laceration*

## Abstract

Open pelvic fractures are rare but represent a serious clinical problem with high mortality rates. Acute mortality is often associated with haemorrhage and delayed mortality is most often associated with sepsis and multiple organ failure. An aggressive multidisciplinary approach is of paramount importance to prevent catastrophe. It involves emergency resuscitation, stabilization of unstable fracture with an external fixator, and faecal diversion for rectal injury. Here, a case of open pelvic fracture with rectal laceration has been presented.

## INTRODUCTION

Open pelvic fractures make up 2-5% of all pelvic ring injuries and their mortality has been reported to be as high as 50%.^1,2^ Mortality in patients with open pelvic fractures is often due to haemorrhage in the acute phase and on a delayed basis, it is usually secondary to sepsis and multiple organ failure.^3,4^

The important elements of successful treatment are emergency resuscitation, fracture stabilization, diverting colostomies for rectal lacerations, and prevention of sepsis.^5,6^ Due to the rare occurrence of the injury, it is very difficult for trauma centres to get extensive experience in determining the best treatment protocol. We report a case of open pelvic fracture with an associated rectal laceration.

## CASE REPORT

A 25 years old male was referred to our centre after 6 hours of injury in the state of hypovolemic shock. He had a road traffic accident and sustained injury over his pelvic region.

At the time of presentation, his general condition was poor. His vitals were unstable. Immediate resuscitation was done as per ATLS guidelines. He had a lacerated wound measuring approximately 15*8 cm over his left groin extending up to the perineum and the left inferior pubic rami bone was exposed. Tetanus prophylaxis and antibiotics were given. A pelvic binder was applied.

**Figure 1 A, B:**
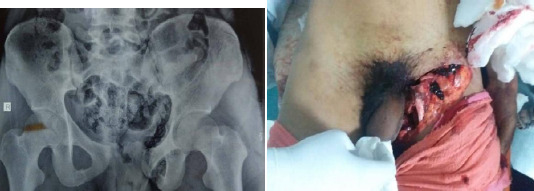
A the time of presentation (A) X-ray anteroposterior view showing pelvic fracture APC Type III (B) Laceration over Left groin.

The X-ray revealed Pelvic fracture APC Type III. CECT showed no active visceral bleeding. After consultation with the general surgery team, the patient was immediately transferred to Operation theatre for emergency surgical intervention. Operative stabilization of the fracture was done with an External fixator with pin insertion in the iliac crest. In the same setting, the surgery team did laparotomy and colostomy for faecal diversion. Lacerated wound infection was controlled with multiple debridements and the use of culturesensitive antibiotics. Delayed wound closure was done.

**Figure 2 A, B:**
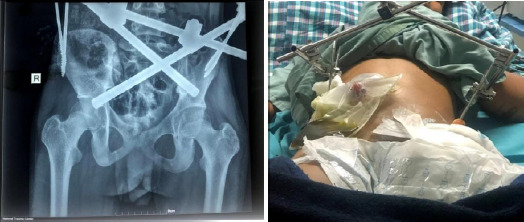
Postoperative status (A) X-ray anteroposterior view (B) External fixator with a colostomy bag.

The external fixator was removed 4 months after the injury. No complications were seen.

**Figure 3:**
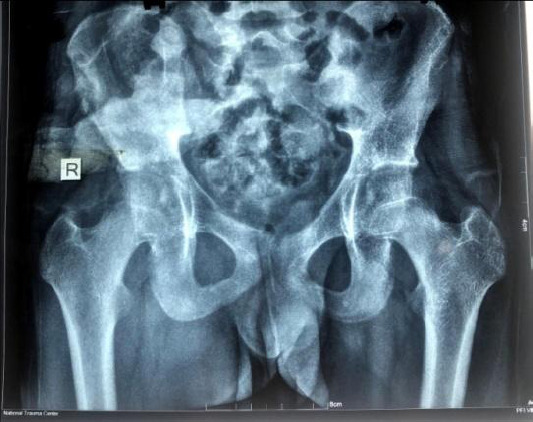
After external fixator removal at 4 months follow-up

## DISCUSSION

Open pelvic fracture is a rare injury with a high mortality rate. With the disruption of the pelvic floor and communication of the retroperitoneal space with the external environment, any potential tamponade is lost, resulting in a massive haemorrhage.^7^ Consequently rapid resuscitation and measures to control haemorrhage in these patients are paramount.

Treatment of unstable open pelvic fracture with associated rectal injury requires an aggressive, multidisciplinary team approach. Jones, et al. looked at 39 patients with open pelvic fractures at two institutions in 10 years and proposed a classification system that divided the injuries into three grades based on the stability of the fracture and whether there was a rectal injury.^7^ They found the association of rectal injury with the development of sepsis to be statistically significant and emphasized the need for diverting colostomy in such patients.

Cannada, et al. did a retrospective review of all patients with an open pelvic fracture at six institutions and found the overall mortality rate to be 23% majority of which being Jones Powell class 3.^5^ They postulated that the use of the pelvic binder, early diverting colostomy might improve the mortality rate.

Hermans, et al. did a study on 24 patients with an open fracture in 10 years and found out the mortality rate to be only 4%.^8^ Compared to other studies, the mortality rate was relatively low the reasons for which were new aggressive trauma protocols including damage control surgery, early faecal diversion, a multidisciplinary team approach, and advances in critical care.

In our case, immediate resuscitation was done at the time of presentation in ER. Emergency operative interventions were done within 24 hours of injury. These factors must be responsible for the good outcome of the patient.

Since open pelvic fracture is a rare injury, there are no studies done in our setting so far and there are no specific treatment protocols. Aggressive resuscitation of a patient, immediate stabilization of unstable fracture, and faecal diversion for rectal laceration is recommended to change this injury from a "killing fracture" to a survivable injury.
